# Supplementary material for: Modeling glioblastoma heterogeneity as a dynamic network of cell states
Source: Mol Syst Biol. 2021 Sep 16;17(9):e10105. doi: 10.15252/msb.202010105 (PMC8444284; doi:10.15252/msb.202010105)
Supplement: Supplementary file 6 — Source Data for Figure 5 [file MSB-17-e10105-s004.zip › Figure5A_sourcedata/GSEA_3017/hallmarks_stateF.GseaPreranked.1621934628066/HALLMARK_HYPOXIA.html]

Details for gene set HALLMARK\_HYPOXIA[GSEA]

|  || Dataset | state33017 |
| Phenotype | NoPhenotypeAvailable |
| Upregulated in class | na\_pos |
| GeneSet | HALLMARK\_HYPOXIA |
| Enrichment Score (ES) | 0.42194033 |
| Normalized Enrichment Score (NES) | 2.1964715 |
| Nominal p-value | 0.0 |
| FDR q-value | 0.0020693142 |
| FWER p-Value | 0.01 |
Table: GSEA Results Summary

  

Fig 1: Enrichment plot: HALLMARK\_HYPOXIA      
 Profile of the Running ES Score & Positions of GeneSet Members on the Rank Ordered List

  

| PROBE | GENE SYMBOL | GENE\_TITLE | RANK IN GENE LIST | RANK METRIC SCORE | RUNNING ES | CORE ENRICHMENT || 1 | CDKN1A |  |  | 4 | 2.089 | 0.0876 | Yes |
| 2 | PNRC1 |  |  | 23 | 1.070 | 0.1252 | Yes |
| 3 | F3 |  |  | 41 | 0.978 | 0.1593 | Yes |
| 4 | NCAN |  |  | 138 | 0.686 | 0.1452 | Yes |
| 5 | HMOX1 |  |  | 142 | 0.684 | 0.1731 | Yes |
| 6 | DTNA |  |  | 176 | 0.617 | 0.1845 | Yes |
| 7 | KLHL24 |  |  | 200 | 0.589 | 0.1993 | Yes |
| 8 | DDIT4 |  |  | 216 | 0.579 | 0.2172 | Yes |
| 9 | RRAGD |  |  | 218 | 0.578 | 0.2415 | Yes |
| 10 | BTG1 |  |  | 222 | 0.574 | 0.2647 | Yes |
| 11 | SLC2A3 |  |  | 228 | 0.569 | 0.2867 | Yes |
| 12 | FAM162A |  |  | 282 | 0.524 | 0.2851 | Yes |
| 13 | BCAN |  |  | 302 | 0.510 | 0.2983 | Yes |
| 14 | IDS |  |  | 307 | 0.507 | 0.3182 | Yes |
| 15 | PAM |  |  | 361 | 0.483 | 0.3149 | Yes |
| 16 | HSPA5 |  |  | 373 | 0.480 | 0.3304 | Yes |
| 17 | ALDOA |  |  | 377 | 0.479 | 0.3495 | Yes |
| 18 | GCNT2 |  |  | 413 | 0.465 | 0.3535 | Yes |
| 19 | PRDX5 |  |  | 419 | 0.463 | 0.3711 | Yes |
| 20 | DDIT3 |  |  | 447 | 0.451 | 0.3781 | Yes |
| 21 | TIPARP |  |  | 472 | 0.444 | 0.3862 | Yes |
| 22 | CA12 |  |  | 522 | 0.426 | 0.3822 | Yes |
| 23 | BNIP3L |  |  | 565 | 0.409 | 0.3807 | Yes |
| 24 | FOS |  |  | 592 | 0.401 | 0.3860 | Yes |
| 25 | IRS2 |  |  | 595 | 0.400 | 0.4022 | Yes |
| 26 | SDC2 |  |  | 596 | 0.399 | 0.4193 | Yes |
| 27 | CHST2 |  |  | 628 | 0.390 | 0.4219 | Yes |
| 28 | VLDLR |  |  | 713 | 0.368 | 0.3996 | No |
| 29 | RORA |  |  | 783 | 0.351 | 0.3834 | No |
| 30 | FOXO3 |  |  | 801 | 0.346 | 0.3905 | No |
| 31 | PPARGC1A |  |  | 821 | 0.343 | 0.3965 | No |
| 32 | VEGFA |  |  | 840 | 0.339 | 0.4029 | No |
| 33 | SDC3 |  |  | 863 | 0.333 | 0.4071 | No |
| 34 | WSB1 |  |  | 882 | 0.329 | 0.4131 | No |
| 35 | BHLHE40 |  |  | 928 | 0.321 | 0.4064 | No |
| 36 | CXCR4 |  |  | 1003 | 0.309 | 0.3861 | No |
| 37 | GPC1 |  |  | 1162 | 0.284 | 0.3266 | No |
| 38 | HK1 |  |  | 1224 | 0.276 | 0.3108 | No |
| 39 | GAPDH |  |  | 1361 | 0.261 | 0.2603 | No |
| 40 | PGM1 |  |  | 1364 | 0.261 | 0.2705 | No |
| 41 | CSRP2 |  |  | 1386 | 0.258 | 0.2721 | No |
| 42 | CITED2 |  |  | 1433 | 0.254 | 0.2621 | No |
| 43 | ANXA2 |  |  | 1511 | -0.263 | 0.2384 | No |
| 44 | ENO1 |  |  | 1673 | -0.308 | 0.1786 | No |
| 45 | UGP2 |  |  | 1725 | -0.327 | 0.1695 | No |
| 46 | SAP30 |  |  | 1826 | -0.378 | 0.1403 | No |
| 47 | LDHA |  |  | 1833 | -0.380 | 0.1539 | No |
| 48 | SERPINE1 |  |  | 2161 | -0.843 | 0.0417 | No |
Table: GSEA details [plain text format]

  

Fig 2: HALLMARK\_HYPOXIA: Random ES distribution      
 Gene set null distribution of ES for **HALLMARK\_HYPOXIA**
